# Potential application of an *Aspergillus* strain in a pilot biofilter for benzene biodegradation

**DOI:** 10.1038/srep46059

**Published:** 2017-04-06

**Authors:** Da Sun, Kun Zhang, Chuanren Duan, Wei Wu, Daiyong Deng, Donghong Yu, M. Babar Shahzad, Dake Xu, Ju Tang, Li Luo, Jia Chen, Jinxuan Wang, Yidan Chen, Xiang Xie, Guixue Wang

**Affiliations:** 1 Key Laboratory for Biorheological Science and Technology of Ministry of Education, State and Local Joint Engineering Laboratory for Vascular Implants, Bioengineering College of Chongqing University, 174 Shazheng Street, Chongqing 400030, China; 2 Institute of Life Sciences, Wenzhou University, Chashan University Town, Wenzhou 325000, China; 3 Department of Chemistry and Environmental Science, New Jersey Institute of Technology, Newark, NJ 07102, USA; 4 Department of Chemistry and Bioscience, Aalborg University, DK-9220, Aalborg, Denmark; 5 Institute of Metal Research, Chinese Academy of Sciences, 72 Wenhua Road, Shenyang 110016, China; 6 Department of Occupational Health, Third Military Medical University, 30 Gaotanyan Street, Chongqing 400038, China

## Abstract

A biofilter with fungus was developed for efficient degradation of benzene, which can overcome the potential risk of leakage commonly found in such services. Results indicated that the optimum parameter values were temperature 40 °C, pH 6, and 500 mg L^−1^ of the initial benzene concentration. Besides, the empty bed residence time and inlet load range of biofilter were set to 20 s and 21.23–169.84 g m^−3^ h^−1^ respectively. Under these conditions, this biofilter can obtain the maximum removal efficiency of more than 90%, the eliminating capacity could be up to 151.67 g m^−3^ h^−1^. Furthermore, scanning electron microscopy was used to investigate three filler materials for packing fungus biofilm. This is the first study introducing an *Aspergillus* strain for benzene removal and these results highlight that the development of this biofilter has the potential scaling-up application as gas-processing of industrial wastes.

Volatile organic compounds (VOCs) are a class of common industrial pollutants mainly consists of benzene, toluene, ethylbenzene, and xylene (BTEX), among which benzene emission playing an extremely important role, and has been a serious threat to the environment and human health^1^,^2^,^3^,^4^. Benzene could be absorbed by bodies via the skin and respiratory tract and could result in pernicious effect on human health, including genotoxicity, hematotoxicity, and leukemia^5^.

For the purpose of metabolizing benzene, catalytic oxidation and intermolecular electron transitions methods have been commonly applied, however, these methods are limited because of their low yields or strict requirement on lights of characteristic wavelengths^6^,^7^,^8^. Recently membrane separation processes have also become restricted due to the high cost^9^,^10^,^11^. Slominska *et al*. regarded plasma methods and biological methods as future trends in the contaminated air purification technology^12^. Recent findings demonstrate that plasma purification method always produces byproducts in process of BTEX degradation, which may cause secondary pollution^13^. Biofilter turns out to be one of the most cost-effective methods to treat the exhaust gas because it has no secondary pollution and especially has superior efficiency in dealing with the low concentration of exhaust gas^14^,^15^.

Suitable microorganisms and filler would not only optimize the biodegrability but also shorten the waste air treatment period. Fungal filament is able to contact with waste gas directly and to decrease mass transfer resistance^16^,^17^. Therefore, biofilters with fungus can withstand harsher conditions than those with bacteria. In spite of its potential leakage risk, security design has received little attention in bioreactor studies.

In this study the microbial strain applied to benzene degradation, namely HD-5, was identified with morphological and molecular identification. In addition, we designed and patented a biofilter (Chinese patent No.: 2005100066284) for gas treatment^18^. Such a biofilter generates enough vacuum through wind cap and solar energy heat-collection plates on top of the biofilter so that gas leakage would be avoided when some cracks appear in the tower body due to the inwards flow of air. The ability to resist impact load and severe environment, and the optimal process conditions of the modified biofilter (Fig. 1) applied with HD-5 strain and fillers were systematically investigated.

## Materials and Methods

### Identification of microorganism

Using benzene (Shenkai gases Technology Co., Shanghai, China) as the sole carbon source, strain HD-5 with effective benzene degrading capability was isolated from activated sludge of sewage treatment plant (Chongqing, China). Strain HD-5 was obtained using centrifugation after being incubated in PDA culture medium for 7 d. A primary morphological observation of HD-5 was performed with an AXIOVERT 35 microscope (Carl Zeiss Jena, Oberkochen, DE). Then the DNA of HD-5 was extracted using the PowerSoil^®^ DNA extraction kit (Mobio Technologies, Carlsbad, CA). A polymerase chain reaction (PCR) reaction was generated with the internal transcribed spacer (ITS) universal primer set ITS1 (5′-TCCGATGGTGAACCTGCGG-3′) and ITS4 (5′-TCCTCCGCTTATTGATATGC-3′)^19^. Reactions contained 1 μL of each primer (10 μmol L^−1^), 12.5 μL 2 × Taq MasterMix (CWBIO, Beijing, China). It was performed using 30 cycles with the following thermocycle profile: initial denaturation at 95 °C for 5 min followed by (95 °C for 30 s, 60 °C for 30 s and 72 °C for 1 min) followed by a final extension at 72 °C for 10 min. Total PCR reaction volume was 25 μL. PCR products were conducted by electrophoresis in a 1% agarose gel. All tested samples were amplified by PCR with ITS universal primers to determine the presence of possible PCR inhibitors in the sample.

These PCR products in agarose were inserted into a pUC19 cloning vector using a simple TA cloning kit (CWBIO, Beijing, China). Clones picked for each of the PCR products were sent to sequencing company (Biomed Co., Beijing, China). Nucleotide sequences were edited and classified, respectively, using the VecScreen of National Center for Biotechnology Information (NCBI; http://www.ncbi.nlm.nih.gov/blast/Blast.cgi) and the Ribosomal Database Project II (RDP; http://rdp.cme.msu.edu/classifier/classifier.jsp)^20^,^21^.

### Parameters optimization of the degradation reaction

Nitrogen gas was purchased from Shenkai gases Technology Co., Ltd. (Shanghai, China). pH (4,6, and 8) and temperature (TEMP; 30, 40, and 50 °C) were used as single factors to investigate the optimum process. Degradation of benzene was studied in a 250 mL Erlenmeyer shake flask, containing 50 mL basal salt medium (Hope Biol-technology, Qingdao, China). The flask was covered with an aluminum foil seal film (QiYi packaging materials, Shanghai, China) to prevent benzene from escaping. Each experiment was replicated three times, and all of the operations were completed in a SWCJIFD super-clean worktable (Antai Airtech Co., Suzhou, China). Two fungal tablets were transferred with ø10 mm stopper borer from PDA plate culture media and inoculated into the Erlenmeyer shake flask. Then shaking culture (180 r min^−1^) for 8 days following the conditions provided in Supplementary Material (Table S1), and the benzene concentration was replicated three times daily.

### Parameters optimization of biofilter

**Figure 1:**
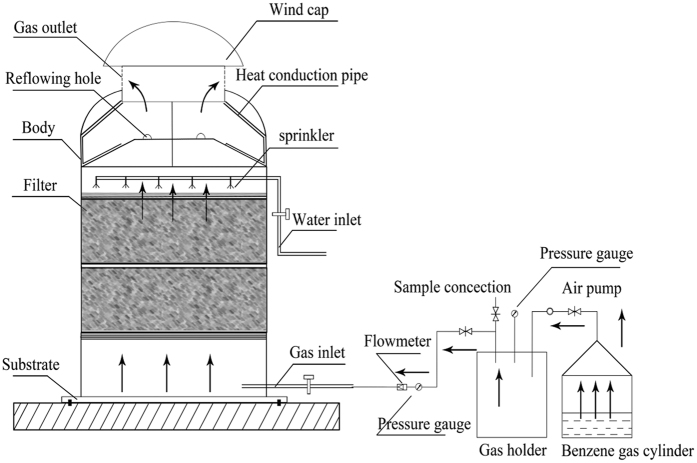
Schematic diagram of biofilter for benzene removal.

As shown in Fig. 1, this tower height was 1000 mm with a diameter of 300 mm. The volume of biofilter was 0.071 m^3^ with a cross-sectional area of 0.071 m^2^. The biofilter was evenly divided into two spaces, each filling layer was 400 mm in height. 500–1000 mL nutrient solution (Table 1) was sprayed each day at 30 °C. Strains HD-5 was injected into biofilter according to the parameters listed in Table 2 with an empty bed residence time (EBRT) of 20 s. The biofilter experienced three periods: low load, intermediate load, and high load operation. The removal efficiency of benzene (*RE*) was calculated based on the following equation:


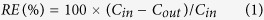


Where *C*_*in*_ and *C*_*out*_ are the benzene concentrations for inlet and outlet respectively.

The following steps were taken to limit the inevitable contamination of the biofilter with other organisms introduced through the waste being treated: (i) HD-5 was inoculated to fillers after the sterilization process of fillers and biofilter. (ii) To ensure that HD-5 has a growth advantage, it will be cultured in sealed overnight.

The concentrations of benzene were measured using a HP6890 GC (Agilent Technologies, Palo Alto, US) with a flame ionization detector and a DB-1 chromatographic column (Model 125-103 J; Agilent Technologies, Palo Alto, US) under the options provided in Supplementary Material (Table S2)^22^.

During biofilm formation (30 d), their surface was observed through a VEGA3SBH scanning electron microscopy (SEM) (Tescan Co., Brno, Czech) at operating voltage of 20 kV. Fillers were picked out after the conclusion of the biofilter run, and then fixed with 2.5% glutaraldehyde. Fillers were then dehydrated with a graded ethanol series of 50%, 75%, 95%, and 100%. The coupons were sputter coated with gold for SEM imaging.

At the end of the treatment, the optimum filler was selected, and the low load period of above-mentioned test was repeated with different EBRTs (10 s,20 s, and 30 s) and inlet loads (21.23, 42.46, 84.92, 169.84, 339.68 and 679.36 g m^−3^ h^−1^). Four criteria for fillers are needed in order to provide good growing conditions for microbes and large-scale application^23^: (1) great surface area; (2) surface hydrophilicity; (3) stable chemical properties; (4) low cost. Thus, coal columnar activated carbon (CCAC), bamboo wood (BW), and biological ceramsite (BC) have been widely applied as fillers of biofilter^24^.

### Quantitative variance of HD-5

Approximately 1 gram each of filler material was milled then put into 9 mL of sterile extraction buffer (0.1% Na_4_P_2_O_7_·10H_2_O and 2% NaCl) and vortexed at maximum speed for 10 minutes. The suspension isolated from solid composition by sedimentation, serially dilution with 0.9% NaCl solution, incubation in a PDA plate for 3 days at 30 ± 2 °C^25^,^26^. The procedure of coating plates was replicated two times.

## Results and Discussion

### Morphological observation and molecular identification

After incubation at 30 °C for 7 days the HD-5 strain colony showed white color as a thin membrane, which had the round morphology with a diameter of 18 mm at first 5 days (Fig. S1), and later on expanded into layer-by-layer rosette patterns and finally formed greenish cottony hyphae. Microscopic image presented the conidial head and conidiophores in scale of hundreds of micrometers, while the diameters of hemispheric acrocyst are 10–20 μm. All these indicate that strain HD-5 is a member of *Moniliales*^27^.

A sequence of ITS region with 567 bp was obtained from sequencing data, the warcup fungal ITS training sets were used to classify fungal ITS sequences which can offer accurate results of classification^28^. The sequence identified with the classifier programme of RDP indicated that the strain HD-5 is *Aspergillus versicolor*. The results obtained by both methods reached a similar conclusion.

### Effects of the pH and TEMP on the benzene removal

**Figure 2:**
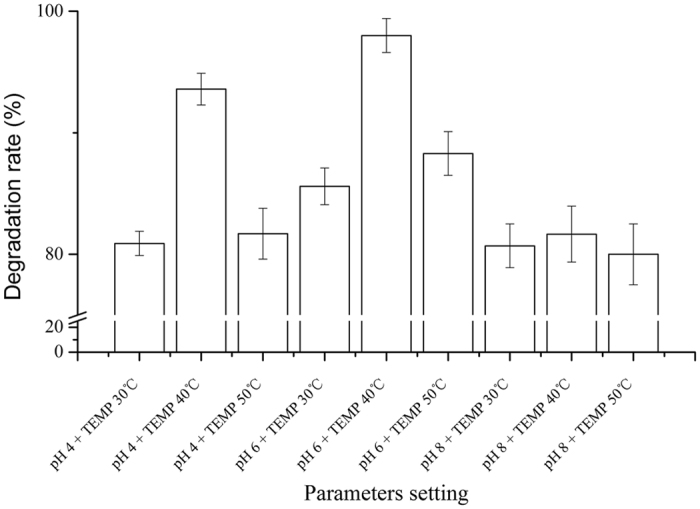
The comparison of benzene removal efficiency with different parameters in 8 d.

Figure 2 exhibits that the removal efficiency reaches maximum value of 98 ± 1.4% when the pH is 6 and the TEMP is 40 °C. it may be due to the enzyme producing ability of *Aspergillus versicolor* achieving the top level under these conditions^29^. As the key enzymes in the biodegradation of benzene series, monooxygenase and dioxygenase appeared in many metabolic pathways of biodegradation^30^,^31^,^32^,^33^. And the removal efficiency became relatively low departing from the optimum reaction TEMP which accorded with the enzymatic properties. pH plays an important role in the secretion of active metabolite. Under abiotic factors duress, the expression of enzyme genes related to benzene metabolism could change accordingly to permit survival of the affected^34^,^35^,^36^. *RE* can exceed 80% in most of the experimental range which may benefit from the resilience of HD-5 to severe environment. Previous studies found that fungus as the biofilm for biofilter has an advantage over bacteria in removing organics^37^,^38^. This advantage cannot be ignored in the practical industry with a great pH fluctuation of industrial wastes^39^,^40^.

### The selection of fillers

**Figure 3:**
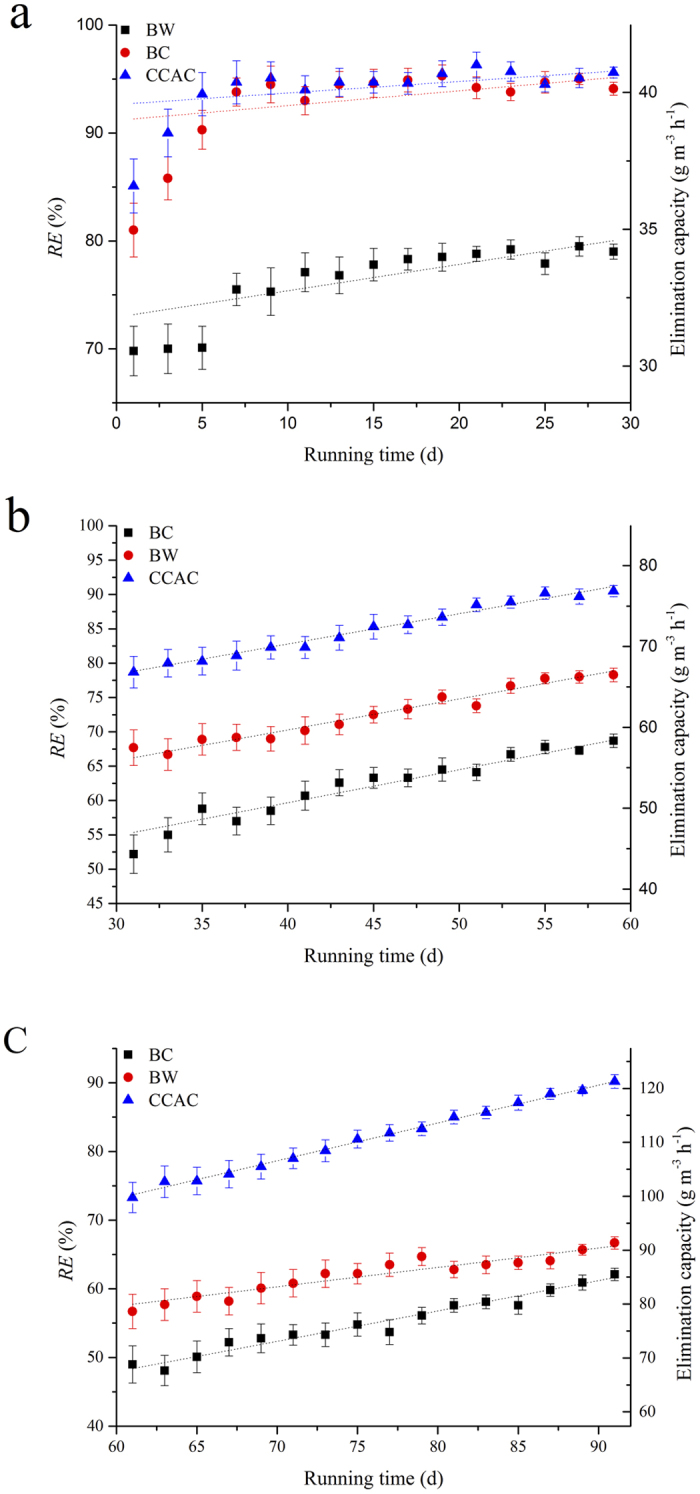
*RE* of biofilters with different fillers in three periods: low load (**a**), intermediate load (**b**) and high load operation (**c**).

**Figure 4:**
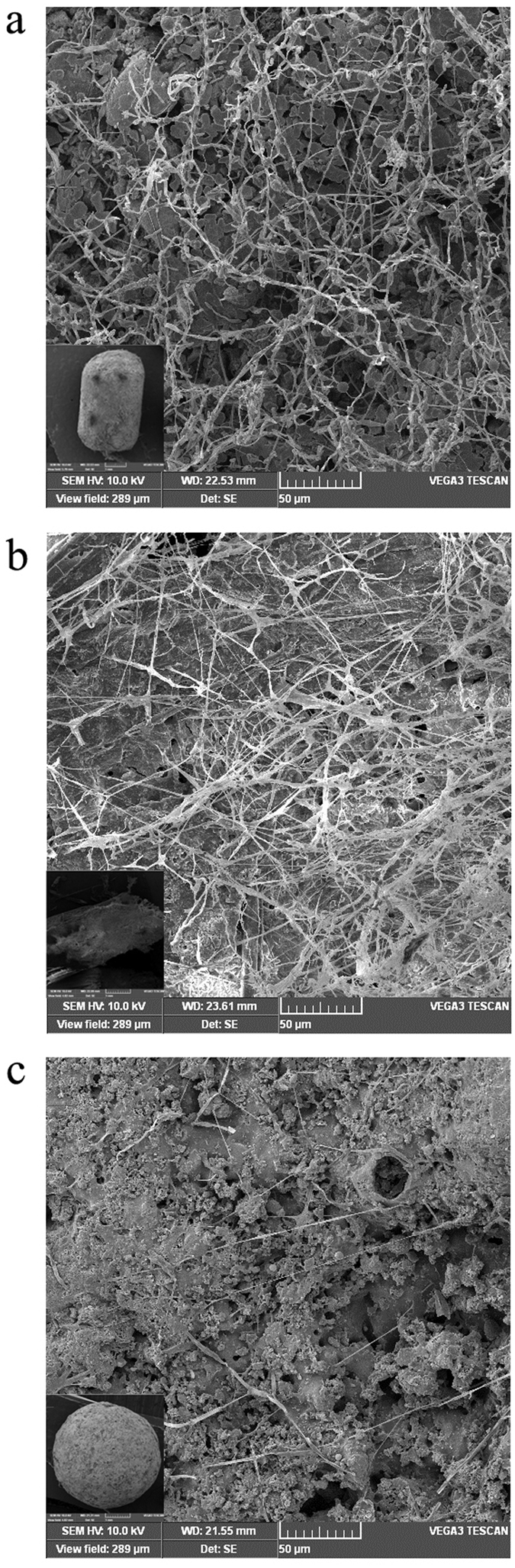
SEM images of biofilm on packing filler: (**a**) CCAC, (**b**) BC, (**c**) BW.

It is evident from Fig. 3 that average *RE (RE*_*ave*_) of CCAC (93.95%) and BC (92.63%) were comparable and significantly superior to BW (76.24%) in the low load. For intermediate and high load operation, *RE*_*ave*_ of CCAC was still the best which can reach 90.5% and 89.3%. Acclimated with metals of low concentration benzene in the initial 30 days, microbes can form biofilms on the surfaces of fillers (Fig. 4). Figure 4 (a,b) illustrates the successful membrane-hanging with good results of acclimation on the CCAC and BC while the surface of BC had less fungal hyphae. It could be due to that CCAC and BC have the larger specific surface areas^41^ which have contributed to the increase of conversion efficiency from gas to liquid or biological phase^42^.

### Effect of inlet load, EBRT and biomass on the benzene removal

**Figure 5:**
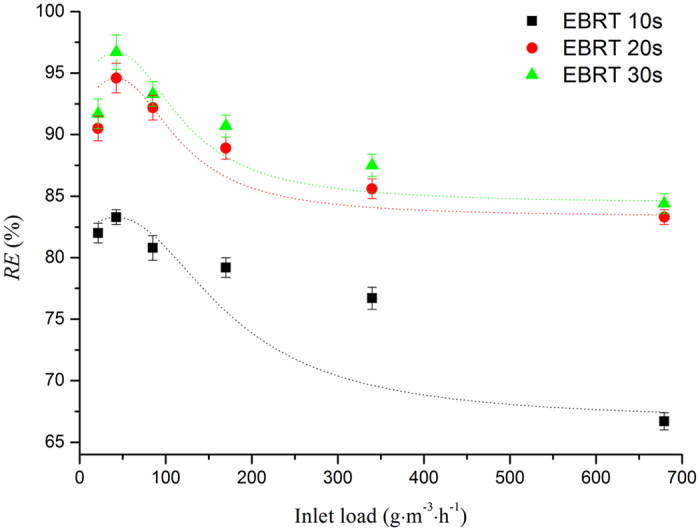
Effect of inlet load and EBRT on benzene removal.

**Figure 6:**
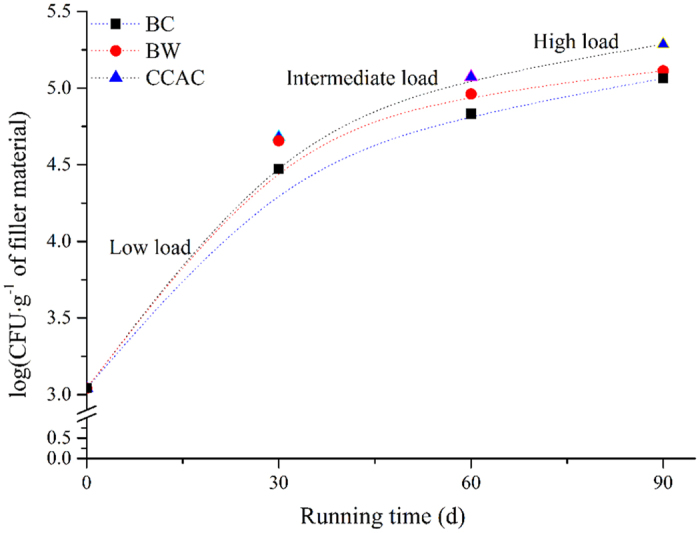
Quantitative variance of HD-5 in the filler materials at the three load periods vs. time.

According to the above analysis, biofilter with CCAC was selected as the focus of further investigation. Under the same EBRT (20 s), the effect of biological benzene removal depends on the quantity and activity of HD-5 in CCAC and biofilm. Figure 5 indicates all *RE* of different EBRT reach the summit when the inlet load was 42.46 g m^−3^ h^−1^. It might be attributed to the relatively few of HD-5 when the inlet load was 21.23 g m^−3^ h^−1^, and the quantity of HD-5 was increased with the inlet load (Fig. 6). Meanwhile the obstacle of mass transfer within the biofilm was triggered by the low diffusive fluxes of benzene, and the biofilm was not fully utilized^43^.

However, *RE* decreased when the inlet load increased to 169.84 g m^−3^ h^−1^, mainly because the HD-5 growth did not keep pace with benzene load (Fig. 6). Besides, matrix suppression effect could also be a major cause^44^,^45^.

Figure 5 Also shows the upside for *RE* is quite limited with EBRT of 20 s which is related directly with operating efficiency and cost. Combined with actual production, the results showed that the ideal EBRT was less than 20 s and that the ideal inlet load is not higher than 169.84 g m^−3^ h^−1^. At last, the results revealed that the performance parameters of biofilter with CCAC and HD-5 have been tremendously strengthened comparing to the biofilters as reported in Table 3.

The present biofilter has simple structure and no need of excessive power source in preventing and eliminating gas leakage effectively. Under windy conditions, the wind cap would be triggered then change the movement of air from original horizontal to vertical direction, thus resulting in a negative pressure. When windless, the heat energy absorbed by solar energy heat-collection plates make a hot-cell occurs between partition and wind cap. The air in the hot-cell rise up so continuously that an airflow from deep to upside happens, thereby the gas leakage could be avoided. In addition, despite efforts to control microbial contamination, there is no guarantee that all contaminations will be complete avoided. Therefore, the future study will include developing qPCR assay or high-throughput sequencing for investigation of the microbial communities’ variation at different operating phases of the biofilter.

## Conclusion

Compared to the biofilters with bacteria, the biofilter applied with HD-5 strain exhibited advantages of high removal efficiency (more than 90%), eliminating capacity (151.67 g m^−3^ h^−1^), and excellent ability to resist impact load and environment. In addition, the results of SEM were consistent with biomass showing that CCAC was a satisfactory filler material for hanging-membrane of fungus. Based on these properties, this biofilter has a spacious application foreground of the large-scale industrial production.

## Additional Information

**How to cite this article**: Sun, D. *et al*. Potential application of an *Aspergillus* strain in a pilot biofilter for benzene biodegradation. *Sci. Rep.* **7**, 46059; doi: 10.1038/srep46059 (2017).

**Publisher's note:** Springer Nature remains neutral with regard to jurisdictional claims in published maps and institutional affiliations.

## Supplementary Material

Supplementary Materials

## Figures and Tables

**Table 1:** Media formulations used in this study.

Nutrient	K_2_HPO_4_	KH_2_PO_4_	^1^NaNO3	MgSO_4_ · 7H_2_O	FeSO_4_ · 7H_2_O
Concentration (mg L^−1^)	627	191	500	48	7.6

**Table 2:** The options of running parameters of biofilter.

Run time (d)	Inlet concentration of benzene (mg m^−3^)	Benzene load of biofilter (gm^−3^ h^−1^)	Gas flow rate (m^3^ h^−1^)
1–30	1000	42.46	2.4
31–60	2000	84.92	2.4
61–90	3000	127.42	2.4

**Table 3:** Performance of biofilters for the removal of benzene as a sole-substrate, as reported in selected previous biofiltration studies, and compared to this study.

Bioreactor/filter media	Inlet concentration (g m^−3^)	Loading rate (g m^−3^ h^−1^)	Removal efficiency (%)	Elimination Capacity (g m^−3^ h^−1^)	Reference
Biofilter/sugarcane bagasse–glass bead mixture	0.01–0.05	6.12	43	3.8	46
Biofilter/sugarcane bagasse	0.4	12	53	6.4	47
Biofilter/peat	0.4	31	84	26	47
Biofilter/granular activated carbon mixture	0.7	21	96	20.1	48
Trickling biofilter/fibrous bed	0.37–1.7	2.6–25.6	>90	11.5	49
Trickling biofilter/coal particles	0.47	64	90	57.6	50
Biofilter/CCAC with HD-5	2	169.84	>90	151.67	This study

## References

[b1] Miller L., Xu X., Grgicak-Mannion A., Brook J. & Wheeler A. Multi-season, multi-year concentrations and correlations amongst the BTEX group of VOCs in an urbanized industrial city. Atmos. Environ. 61, 305–315 (2012).

[b2] An T. . Pollution profiles and health risk assessment of VOCs emitted during e-waste dismantling processes associated with different dismantling methods. Environ. Int. 73, 186–194 (2014). 25129414 10.1016/j.envint.2014.07.019

[b3] Dinh T.-V. . Emission characteristics of VOCs emitted from consumer and commercial products and their ozone formation potential. Environ. Sci. Pollut. Res. 22, 9345–9355 (2015). 10.1007/s11356-015-4092-8 25601614

[b4] Mitri S. . Metabolic polymorphisms and clinical findings related to benzene poisoning detected in exposed Brazilian gas-station workers. Int. J. Environ. Res. Public. Health 12, 8434–47 (2015). 26197327 10.3390/ijerph120708434 PMC4515729

[b5] Krieg E. F. . Detection of DNA damage in workers exposed to JP-8 jet fuel. Mutat. Res.-Genet. Toxicol. Environ. Mutagen. 747, 218–227 (2012). 10.1016/j.mrgentox.2012.05.005 22617435

[b6] Hinojosa-Reyes M., Rodriguez-Gonzalez V. & Arriaga S. Enhancing ethylbenzene vapors degradation in a hybrid system based on photocatalytic oxidation UV/TiO2-In and a biofiltration process. J. Hazard. Mater. 209, 365–371 (2012). 22296707 10.1016/j.jhazmat.2012.01.035

[b7] Joung H.-J. . Catalytic oxidation of VOCs over CNT-supported platinum nanoparticles. Appl. Surf. Sci. 290, 267–273 (2014).

[b8] Pham T. & Lee B. Novel adsorption and photocatalytic oxidation for removal of gaseous toluene by V-doped TiO/PU under visible light. J. Hazard. Mater. 30, 493–503 (2015). 10.1016/j.jhazmat.2015.07.048 26247377

[b9] Trusek-Holownia A. & Noworyta A. Advanced treatment of wastewater with BTEX. Desalination Water Treat 50, 440–445 (2012).

[b10] Liu Y. & Shi B. Interaction parameter method for investigating BTEX extraction using the hollow-fiber microporous membrane liquid/liquid extraction technique. Chem. Eng. Technol. 32, 926–931 (2009).

[b11] Yahaya G. O. Separation of volatile organic compounds (BTEX) from aqueous solutions by a composite organophilic hollow fiber membrane-based pervaporation process. J. Membr. Sci. 319, 82–90 (2008).

[b12] Slominska M., Krol S. & Namiesnik J. Removal of BTEX compounds from waste gases; destruction and recovery techniques. Crit. Rev. Environ. Sci. Technol. 43, 1417–1445 (2013).

[b13] Kuroki T., Hirai K., Kawabata R., Okubo M. & Yamamoto T. Decomposition of adsorbed xylene on adsorbents using nonthermal plasma with gas circulation. Ieee Trans. Ind. Appl. 46, 672–679 (2010).

[b14] Chen X., Qian W., Kong L., Xiong Y. & Tian S. Performance of a suspended biofilter as a new bioreactor for removal of toluene. Biochem. Eng. J. 98, 56–62 (2015).

[b15] Rene E. R. . Start-up, performance and optimization of a compost biofilter treating gas-phase mixture of benzene and toluene. Bioresour. Technol. 190, 529–535 (2015). 25827361 10.1016/j.biortech.2015.03.049

[b16] Spigno G., Pagella C., Fumi M. D., Molteni R. & De Faveri D. M. VOCs removal from waste gases: gas-phase bioreactor for the abatement of hexane by *Aspergillus niger*. Chem. Eng. Sci. 58, 739–746 (2003).

[b17] van Groenestijn J. W. & Liu J. X. Removal of alpha-pinene from gases using biofilters containing fungi. Atmos. Environ. 36, 5501–5508 (2002).

[b18] Sun D., Duan C., Liu A., Huang Y. & Bao J. Patent center of Chongqing University. A biological filter. Chinese patent CN 2,012,200,181,465. 2012 Jan 16.

[b19] WHITE T. Analysis of phylogenetic relationships by amplification and direct sequencing of ribosomal RNA genes. PCR Protoc. Guide Methods Appl. doi: 10.1007/978-94-011-4020-1_3 (1990).

[b20] Cole J. R. The Ribosomal Database Project (RDP-II): sequences and tools for high-throughput rRNA analysis. Nucleic Acids Res. 33, D294–D296 (2004). 10.1093/nar/gki038 PMC539992 15608200

[b21] Sun D. . Application of *Faecalibacterium* 16S rDNA genetic marker for accurate identification of duck faeces. Environ. Sci. Pollut. Res. 23, 7639–7647 (2016). 10.1007/s11356-015-6024-z 26743644

[b22] Jiang C. . Enhanced butanol production through adding organic acids and neutral red by newly isolated butanol-tolerant bacteria. Appl. Biochem. Biotechnol. 180, 1–12 (2016). 27351984 10.1007/s12010-016-2176-7

[b23] Álvarez-Hornos F. J. . Biofiltration of ethylbenzene vapours: Influence of the packing material. Bioresour. Technol. 99, 269–276 (2008). 17317157 10.1016/j.biortech.2006.12.022

[b24] Wang G. . Adsorption of benzene, cyclohexane and hexane on ordered mesoporous carbon. J. Environ. Sci.-China 30, 65–73 (2015). 25872710 10.1016/j.jes.2014.10.015

[b25] Saravanan V. & Rajamohan N. Treatment of xylene polluted air using press mud-based biofilter. J. Hazard. Mater. 162, 981–988 (2009). 18632206 10.1016/j.jhazmat.2008.05.158

[b26] Rene E. R., Murthy D. V. S. & Swaminathan T. Steady- and transient-state effects during the biological oxidation of gas-phase benzene in a continuously operated biofilter. Clean Technol. Environ. Policy 12, 525–535 (2010).

[b27] Barnett H. L. & Hunter B. B. Illustrated genera of imperfect fungi. (APS Press, 1998).

[b28] Wang Q., Garrity G. M., Tiedje J. M. & Cole J. R. Naive Bayesian classifier for rapid assignment of rRNA sequences into the new bacterial taxonomy. Appl. Environ. Microbiol. 73, 5261–5267 (2007). 17586664 10.1128/AEM.00062-07 PMC1950982

[b29] Andrade S. D., Polizeli M., Terenzi H. F. & Jorge J. A. Effect of carbon source on the biochemical properties of beta-xylosidases produced by *Aspergillus versicolor*. Process Biochem. 39, 1931–1938 (2004).

[b30] Lovley D., Coates J., Woodward J. & Phillips E. Benzene oxidation coupled to sulfate reduction. Appl. Environ. Microbiol. 61, 953–958 (1995). 16534979 10.1128/aem.61.3.953-958.1995 PMC1388378

[b31] Heider J. & Fuchs G. Microbial anaerobic aromatic metabolism. Anaerobe 3, 1–22 (1997). 16887557 10.1006/anae.1997.0073

[b32] Hendrickx B. . PCR-DGGE method to assess the diversity of BTEX mono-oxygenase genes at contaminated sites. Fems Microbiol. Ecol. 55, 262–273 (2006). 16420634 10.1111/j.1574-6941.2005.00018.x

[b33] Hendrickx B. . Alternative primer sets for PCR detection of genotypes involved in bacterial aerobic BTEX degradation: Distribution of the genes in BTEX degrading isolates and in subsurface soils of a BTEX contaminated industrial site. J. Microbiol. Methods 64, 250–265 (2006). 15949858 10.1016/j.mimet.2005.04.018

[b34] Lopez-Malo A., Alzamora S. M. & Palou E. *Aspergillus flavus* growth in the presence of chemical preservatives and naturally occurring antimicrobial compounds. Int. J. Food Microbiol. 99, 119–128 (2005). 15734560 10.1016/j.ijfoodmicro.2004.08.010

[b35] LopezMalo A., Alzamora S. M. & Argaiz A. Effect of vanillin concentration, pH and incubation temperature on *Aspergillus flavus, Aspergillus niger, Aspergillus ochraceus* and *Aspergillus parasiticus* growth. Food Microbiol. 14, 117–124 (1997).

[b36] Redkar R. J., Locy R. D. & Singh N. K. Biosynthetic pathways of glycerol accumulation under salt stress in *Aspergillus nidulans*. Exp. Mycol. 19, 241–246 (1995). 8574901 10.1006/emyc.1995.1030

[b37] Maestre J. P., Gamisans X., Gabriel D. & Lafuente J. Fungal biofilters for toluene biofiltration: Evaluation of the performance with four packing materials under different operating conditions. Chemosphere 67, 684–692 (2007). 17184815 10.1016/j.chemosphere.2006.11.004

[b38] Garcia-Pena I., Ortiz I., Hernandez S. & Revah S. Biofiltration of BTEX by the fungus *Paecilomyces variotii*. Int. Biodeterior. Biodegrad. 62, 442–447 (2008).

[b39] Valdez-Vazquez I., Rios-Leal E., Carmona-Martinez A., Munoz-Paez K. M. & Poggi-Varaldo H. M. Improvement of biohydrogen production from solid wastes by intermittent venting and gas flushing of batch reactors headspace. Environ. Sci. Technol. 40, 3409–3415 (2006). 16749714 10.1021/es052119j

[b40] Baawain M. S., Al-Jabri M. & Choudri B. S. Characterization of industrial wastewater sludge in Oman from three different regions and recommendations for alternate reuse applications. Iran. J. Public Health 44, 1473–1481 (2015). 26744704 PMC4703226

[b41] Ducman V. & Mirtic B. The applicability of different waste materials for the production of lightweight aggregates. Waste Manag. 29, 2361–2368 (2009). 19345083 10.1016/j.wasman.2009.02.013

[b42] Pedersen L.-F., Pedersen P. B. & Sortkjaer O. Temperature-dependent and surface specific formaldehyde degradation in submerged biofilters. Aquac. Eng. 36, 127–136 (2007).

[b43] Singh K., Singh R. S., Rai B. N. & Upadhyay S. N. Biofiltration of toluene using wood charcoal as the biofilter media. Bioresour. Technol. 101, 3947–3951 (2010). 20137916 10.1016/j.biortech.2010.01.025

[b44] Dorado A. D., Baeza J. A., Lafuente J., Gabriel D. & Gamisans X. Biomass accumulation in a biofilter treating toluene at high loads - Part 1: Experimental performance from inoculation to clogging. Chem. Eng. J. 209, 661–669 (2012).

[b45] Gutierrez-Acosta O. B., Arriaga S., Escobar-Barrios V. A., Casas-Flores S. & Almendarez-Camarillo A. Performance of innovative PU-foam and natural fiber-based composites for the biofiltration of a mixture of volatile organic compounds by a fungal biofilm. J. Hazard. Mater. 201, 202–208 (2012). 22178276 10.1016/j.jhazmat.2011.11.068

[b46] Sene L., Converti A., Felipe M. G. A. & Zilli M. Sugarcane bagasse as alternative packing material for biofiltration of benzene polluted gaseous streams: a preliminary study. Bioresour. Technol. 83, 153–157 (2002). 12056491 10.1016/s0960-8524(01)00192-4

[b47] Zilli M., Daffonchio D., Di Felice R., Giordani M. & Converti A. Treatment of benzene-contaminated airstreams in laboratory-scale biofilters packed with raw and sieved sugarcane bagasse and with peat. Biodegradation 15, 87–96 (2004). 15068370 10.1023/b:biod.0000015613.91044.a4

[b48] Kim J. O. Degradation of benzene and ethylene in biofilters. Process Biochem. 39, 447–453 (2003).

[b49] Zhou Q., Huang Y. L., Tseng D. H., Shim H. & Yang S. T. A trickling fibrous-bed bioreactor for biofiltration of benzene in air. J. Chem. Technol. Biotechnol. 73, 359–368 (1998).

[b50] Lu C. Y., Chu W. C. & Lin M. R. Removal of BTEX vapor from waste gases by a trickle bed biofilter. J. Air Waste Manag. Assoc. 50, 411–417 (2000). 10734712 10.1080/10473289.2000.10464021

