# Supplementary material for: Potential application of an Aspergillus strain in a pilot biofilter for benzene biodegradation
Source: Sci Rep. 2017 Apr 6;7:46059. doi: 10.1038/srep46059 (PMC5382587; doi:10.1038/srep46059)
Supplement: Supplementary Materials [file srep46059-s1.doc]

**Supplementary Material**

# Potential application of an *Aspergillus* strain in a pilot biofilter for benzene biodegradation

Da Sun^1^ †, Kun Zhang^1^ †, Chuanren Duan^1^ † *, Wei Wu^1^, Daiyong Deng^2^, Donghong Yu^1, 3^, M. Babar Shahzad^4^, Dake Xu^4^, Ju Tang^5^, Li Luo^1^, Jia Chen^1^, Jinxuan Wang^1^, Yidan Chen^1^, Xiang Xie^1^, Guixue Wang^1^ *

### *^1^ Key Laboratory for Biorheological Science and Technology of Ministry of Education, State and Local Joint Engineering Laboratory for Vascular Implants, Bioengineering College of* *Chongqing University,* *174 Shazheng Street, Chongqing 400030, China*

*^2^* *Department of Chemistry and Environmental Science, New Jersey Institute of Technology,* *Newark, NJ 07102, USA*

*^3^ Department of Chemistry and Bioscience, Aalborg University, DK-9220, Aalborg, Denmark*

### *^4^ Institute of Metal Research, Chinese Academy of Sciences, 72 Wenhua Road, Shenyang 110016, China*

### *^5^ Department of Occupational Health, Third Military Medical University, 30 Gaotanyan Street, Chongqing 400038, China*

### ^†^ These authors contributed equally to this work.

*Corresponding authors: guixue_wang@126.com (G.X. Wang); [chrduan@cqu.edu.cn](mailto:chrduan@cqu.edu.cn) (C.R. Duan).

**Lists of Tables**

**Table S1** Orthogonal test designs table of L9 (2^3^)

**Table S2** The options of running parameters of GC

**Table S1** Orthogonal test designs table of L9 (2^3^)

| Test number | Parameters setting | |
| --- | --- | --- |
|  | pH | Temperature (°C) |
| 1 | 4 | 30 |
| 2 | 4 | 40 |
| 3 | 4 | 50 |
| 4 | 6 | 30 |
| 5 | 6 | 40 |
| 6 | 6 | 50 |
| 7 | 8 | 30 |
| 8 | 8 | 40 |
| 9 | 8 | 50 |

**Table S2 The options of running parameters of GC**

| Carrier gas | Flow rate  (mL min^-1^) | Detection Tm | Column Tm (°C) | Vaporization Tm (°C) | Injection volume (μL) |
| --- | --- | --- | --- | --- | --- |
| Nitrogen | 80 | 240 | 80 | 200 | 100 |

**List of Figures**

**Figure S1** Colonies morphology of strains HD-5 on PDA plate culture media cultured at 30°C for 7 days


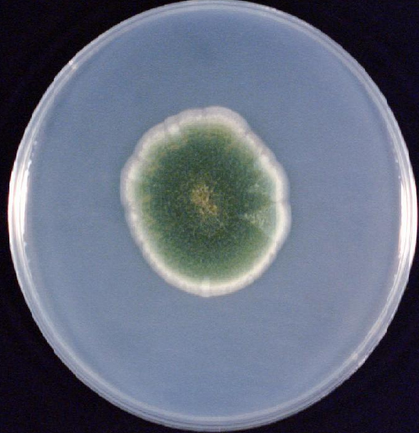


**Figure S1** Colonies morphology of strains HD-5 on PDA plate culture media cultured at 30 °C for 7 days
